# Lipid Metabolism in Development and Progression of Hepatocellular Carcinoma

**DOI:** 10.3390/cancers12061419

**Published:** 2020-05-31

**Authors:** Moris Sangineto, Rosanna Villani, Francesco Cavallone, Antonino Romano, Domenico Loizzi, Gaetano Serviddio

**Affiliations:** 1 Centro Universitario per la Ricerca e la Cura delle Epatopatie (C.U.R.E.), Liver Unit, Università di Foggia, 71100 Foggia, Italy; rosanna.villani@unifg.it (R.V.); francescocavallone87@gmail.com (F.C.); g.serviddio@unifg.it (G.S.); 2 Institute of Internal Medicine, Università di Foggia, 71100 Foggia, Italy; dott.romano@gmail.com; 3 Institute of Thoracic Surgery, Università di Foggia, 71100 Foggia, Italy; domenico.loizzi@unifg.it

**Keywords:** hepatocellular carcinoma, lipid metabolism, fatty acid β-oxidation, lipidomics, tumour progression, non-alcoholic fatty liver disease

## Abstract

Metabolic reprogramming is critically involved in the development and progression of cancer. In particular, lipid metabolism has been investigated as a source of energy, micro-environmental adaptation, and cell signalling in neoplastic cells. However, the specific role of lipid metabolism dysregulation in hepatocellular carcinoma (HCC) has not been widely described yet. Alterations in fatty acid synthesis, β-oxidation, and cellular lipidic composition contribute to initiation and progression of HCC. The aim of this review is to elucidate the mechanisms by which lipid metabolism is involved in hepatocarcinogenesis and tumour adaptation to different conditions, focusing on the transcriptional aberrations with new insights in lipidomics and lipid zonation. This will help detect new putative therapeutic approaches in the second most frequent cause of cancer-related death.

## 1. Introduction

In the last few decades, the incidence of hepatocellular carcinoma (HCC) has rapidly increased and HCC has become the second most frequent cause of cancer-related death worldwide [1,2]. The short-term prognosis has improved because of new advances in therapy and early diagnosis, although the long term prognosis remains poor, with a 5-year survival rate of 17% [3,4,5]. The surgical resection is still the option with the highest recovery rate; however, only 15% of patients are eligible and the 5-year recurrence rate is about 70% [6,7]. Therefore, new pharmacological treatment options are needed. More than 90% of liver cancers develop in chronic liver disease, typically associated with viral infections, such as hepatitis B virus (HBV) and hepatitis C virus (HCV) [8]. However, the incidence of non-viral HCC is increasing, since obesity, diabetes, and alcohol drinking in young people are a real outbreak [9,10,11]. It is indeed estimated that, by 2030, in US, the number of non-alcoholic fatty liver disease (NAFLD) and non-alcoholic steato-hepatitis (NASH) cases will increase about 21% and 63%, respectively, while HCC cases will increase by 137% [9]. Viral and non-viral aetiologies normally encompass a spectrum of histological alterations from simple steatosis to inflammation, fibrosis with cirrhosis, and complications, such as HCC. However, obesity, diabetes, and steatosis account for independent risk factors of HCC [12,13,14], which can indeed occur in early stages of liver diseases, especially in metabolic syndrome. Recently, cancer research has been focused on the role of metabolic reprogramming in tumours, particularly on the role of lipid metabolism. The recent advances in the understanding of the role of lipids in HCC provide a new point of view for the interpretation of tumour development and progression. HCC pathogenesis is very complex as several factors are involved, such as cytokine pattern, ER stress, insulin resistance, oxidative stress, gut microbiota [15,16,17,18,19,20,21], and genetics. In the present review, we shed light on metabolic lipid alterations as a cornerstone in hepatocellular tumour initiation and adaptation.

### 1.1. Lipid Metabolism in HBV- and HCV-Related HCC

A milestone paper that first established the association between HBV infection and HCC in the Taiwanese population was published in 1981 [22]. Over 90% of HBV cases resolve spontaneously, but 25% of chronic infections can evolve into HCC [23,24]. In East Asian countries, the HCC incidence rates were estimated in a systematic review, reporting 0.2 person-years per 100 person-years in inactive carriers, 0.6 person-years in chronic HBV infection without cirrhosis, and 3.7 person-years in compensated cirrhosis [25]. Interestingly, although 70–90% of HBV-related HCC occurs in cirrhotic patients, cases in absence of cirrhosis are also possible [26]. Several studies have described lipid metabolism alterations in HBV infections [27,28,29]. Park et al. reported the hepatic suppression of choline-phosphate cytidyltransferase A (PCYT1A) expression in HBV infected mice, with significant differences in phosphatidylcholine composition [30]. Accordingly, Li et al. showed the up-regulation of phosphatidylcholine biosynthesis promoted by choline kinase alpha (CHKA) in HepG2 cells, a pathway required for HBV replication [31]. In metabolomic and gene expression analysis, Teng et al. used transgenic mice expressing the HBx gene of HBV (HBx mice) to investigate the lipid profile in serum and liver during tumorigenesis [32]. They identified two peaks, one related to generic inflammation and oxidative stress and the second corresponding to the tumour phase after a resolution phase. The tumour phase correlated with the expression of five genes implied in lipid metabolism: arachidonate 5-lipoxygenase, lipoprotein lipase, fatty acid binding protein 4, 1-acylglycerol-3-phosphate O-acyltransferase 9, and apolipoprotein A-IV. The same data were later validated both in vitro and in human HBV-related HCC. Overall, these studies suggest a potential role of lipid metabolism as a driving force in HBV-related HCC.

HCV has been so far the most frequent cause of virus related HCC [6]. Recent direct-acting antivirals cure almost 100% of HCV infections, predicting an important reduction of future infections and HCV-related liver disease. The high cost of such drugs will impact the world epidemiology of HCV-related HCC with significant reduction in western countries, but not in developing areas. Moreover, infected people still have risk the development of HCC, despite the availability of an effective therapy [33,34]. In a recent study, we showed that during viral eradication with direct-acting antivirals (DAAs), vascular endothelial growth factor (VEGF) circulating levels increase until the end of the treatment in a mutating inflammatory background, accounting VEGF as a reasonable tumoral risk factor [35]. In accordance with these observations, HCV-related HCC incidence is predicted to increase until 2030, despite the new therapeutic approaches [36]. It has been widely proved that HCV replication interferes with cell survival and proliferation and with gene expression [37,38]; nevertheless, the pathogenesis of HCV-induced HCC is still largely unclear. The association of HCV infection with steatosis and diabetes has emerged powerfully in the last few years. HCV replication induces hepatic steatosis through insulin resistance or by direct metabolic interference of core proteins [39]. HCV proteins can inhibit microsomal transfer protein activity, an enzyme involved in the formation and secretion of very low density lipoprotein (VLDL) and, therefore, with consequent accumulation of triglycerides (TAG) [40,41]. Moreover, as shown in human tissue, animal model, and cell lines, the regulatory element binding protein (SREBP-1c) is up-regulated with consequent transcriptional activation of enzymes involved in fatty acid (FA) synthesis, such as acetyl-CoA carboxylase (ACC), sterol CoA dehydrogenase 4 (SCD4), and fatty acid synthase (FASN) [42,43,44]. Lerat et al. reported that transgenic mice with hepatic expression of HCV proteins are characterised by high lipogenesis and defective TAG exportation with final micro- and macro-vesicular steatosis [45]. Furthermore, males are particularly inclined to develop HCC. Several of these mechanisms are typically involved in lipid metabolic reprogramming of liver carcinogenesis, as discussed below. Therefore, we can assume that HCV-related lipid perturbations might play a key role in HCC development.

### 1.2. NAFLD and HCC

The incidence of non-viral related HCC is increasing, particularly in developed countries where vaccination for HBV and DAAs for HCV are constantly changing the scenario. Most of these patients are affected by steatohepatitis or cirrhosis [19,46]. However, even NAFLD can directly evolve to HCC independently of NASH [13,47,48]. In fact, being overweight alone increases the risk of HCC by 17%, while obesity increases the risk by 89% [49]. In addition, in patients with viral hepatitis (HBV and HCV), obesity was associated with a higher risk of HCC, highlighting a synergistic pro-tumoral effect provoked by dysregulation of lipid metabolism [46,50]. Even if the mechanisms by which obesity and steatosis promote hepatic carcinogenesis remain quite unclear, the role of lipid dysregulation in this process is well recognized. Since the pathogenesis of HCC in metabolic syndrome is complex, we should consider interaction among multiple patterns, as recently defined in the multiple hit model [51]. For example, during obesity development, the liver accumulates fat to counteract excess of free FAs [52,53], which in turn induces the release of gut-derived endotoxins and the production of adipose tissue cytokines, alongside the worsening of liver inflammation and damage [20,54,55,56,57,58].

### 1.3. Alcohol and HCC

Alcohol-related liver disease (ALD) includes a wide spectrum of histological hepatic alterations, such as steatosis, alcoholic hepatitis (AH), cirrhosis, and HCC [59]. In western countries, ethanol still represents the main cause of liver disease [60,61]. Ethanol provokes liver damage by multiple mechanisms, which include oxidative stress, lipids accumulation, inflammation, and leaky gut [62]. Interestingly, histological features typically observed in ALD depend on lipid accumulation; in fact, ethanol promotes FA synthesis and suppresses oxidation. On the other hand, ethanol increases hepatic uptake of FAs, which will be incorporated in TAG [63,64,65] by the up-regulation of de novo lipogenesis genes, such as ACC1, FASN, and SCD1 via sterol regulatory element-binding protein1 (SREBP1c), as described in different murine models [66,67,68]. Several findings in cultured hepatocytes and murine livers report that ethanol is also able to over-express Lipin-1, a protein with Mg2+-dependent phosphatidic acid phosphohydrolase (PAP) activity, involved in the penultimate step of triglyceride synthesis [69,70,71,72,73,74]. Despite the high availability of FAs in alcohol drinking, β-oxidation is dampened. This effect seems to depend on peroxisome proliferator activated receptor alpha (PPAR-α) inhibition, as observed in vitro and in animal models [75]. Accordingly, in ethanol-fed mice, PPARα DNA binding activity decreases with consequent lower expression of enzymes essential in fatty acids oxidation (FAO) [76].

## 2. Lipid Metabolic Reprogramming

Carcinogenesis and tumour adaptation to a local microenvironment are fuelled by metabolic alterations, which promote the survival of cancer cells. This process is known as metabolic reprogramming, a set of adaptations clonally selected during tumorigenesis by generating metabolites with different functions at different levels [77]. The two most studied examples of tumoral metabolic reprogramming are the Warburg effect and glutaminolysis [78]. Reprogramming cell energetic functions is an important survival strategy in cancer, as shown by the enhancement of aerobic glycolysis instead of mitochondrial oxidative phosphorylation (the Warburg effect) [79]. Similarly, the increased glutamine metabolism, glutaminolysis, sustains the mitochondrial tricarboxylic acid (TCA) cycle by generation of citrate and αketoglutarate [80]. Moreover, the role of lipid metabolism in cancer is receiving more and more attention, and thus, several studies show that FAs and/or cholesterols promote tumoral activity [81,82,83,84,85,86]. The multiple functions of lipids in cell signalling, membrane components, and sources of energy are essential in cancer cells [87]. The high “starvation” of lipids in tumours is fulfilled by external uptake and de novo lipogenesis [88], and, consequently, FAO is also increased in several tumour types [89,90].

**Figure 1:**
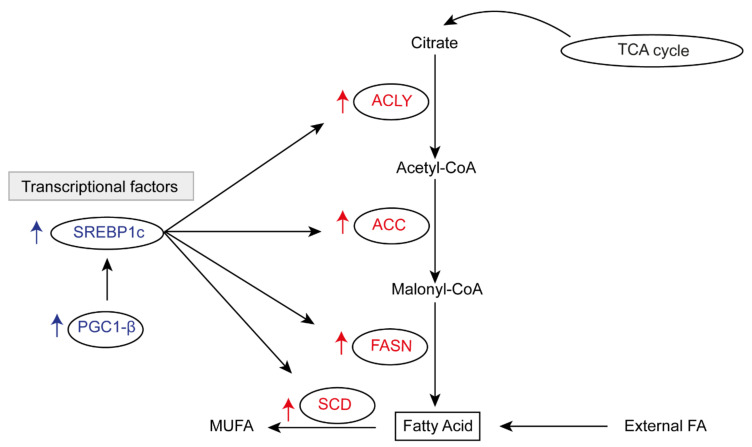
Enhancement of fatty acid synthesis in hepatocellular carcinoma (HCC). ATP citrate lyase (ACLY); acetyl-CoA carboxylase (ACC); fatty acid synthase (FASN); stearoyl-CoA desaturase (SCD); fatty acid (FA); monounsaturated fatty acid (MUFA); tricarboxylic acid cycle (TCA cycle); sterol regulatory element-binding protein1 (SREBP1c); peroxisome proliferator-activated receptor-γ coactivator beta (PGC-1β).

HCC is typically characterised by up-regulation of genes involved in FA synthesis, such as ATP-citrate lyase (ACLY), acetyl-CoA carboxylase (ACC), and fatty acid synthase (FASN), which induce conversion of citrate to acetyl-CoA, malonyl-CoA, and FA, respectively [91,92,93,94,95,96,97,98]. FAs are converted to monounsaturated fatty acids (MUFA); sources for the synthesis of TAG. Stearoyl-CoA desaturase (SCD) is responsible for MUFA generation, and its up-regulation has been associated with HCC in humans [88]. Moreover, the expression of these genes involved in FA synthesis can be regulated by the transcription factor SREBP-1c, whose lipogenic pathway is upregulated in human HCC [99]. Interestingly, hepatic lipogenesis is further modulated by peroxisome proliferator-activated receptor-γ coactivator β (PGC-1β), a transcriptional cofactor which interacts with SREB1c, inducing the transcription of genes, such as as FASN and SCD1 [100] (Figure 1). PGC-1β overexpression in mice promotes HCC development [101]. 

However, the de novo lipogenesis is not a unique source of FAs in HCC. In obesity- and NASH-related HCC, the persistent lipolysis of the adipose tissue provides an enormous amount of FAs (in particular non-esterified FAs) to the liver, with consequent adaptation to this stress [102]. To do this, the liver increases aerobic glycolysis and glutamine synthesis with the enhancement of TCA, promoting hepatocarcinogenesis [103]. However, the mechanism to explain how malignant cells escape lipotoxicity is not yet understood. Recent studies described a new histological variant of HCC, steatohepatitic HCC (SH-HCC), whose peculiarity is macrovesicular steatosis with underlying viral or non-viral steatohepatitis [104,105]. Accordingly, most obesity-related HCC models in mice are characterised by a prominent lipid accumulation in tumoral tissue than in non-tumoral tissue [19,20,106]. Fujiwara et al. suggested that this is plausible because, in the diethylnitrosamine (DEN)-induced HCC model, high fat diet (HFD)-fed mice showed an up-regulation of CPT1A, which converts FA-derived acyl-CoA to Acylcarnitine, while CPT2 (which reconverts acylcarnitine to AcylCoA) results in down-regulated CPT2 [107]. The consequence is the accumulation of Acylcarnitine with oncogenic effects and lower availability of acyl-CoA for β oxidation, resulting in lipids storage. In human SH-HCC, CPT2 expression is also downregulated and high circulating levels of acylcarnitine are detectable in subjects with NASH or HCC [107,108]. Similar results in terms of gene expression profile are given in other murine models, such as major urinary protein (MUP)-urokinase-type plasminogen activator (MUP-uPA) mice [19] and phosphatidylinositol-4,5-bisphosphate 3-kinase catalytic subunit alpha (PIK3CA) transgenic mice [106]. Collectively, these works show how CPT2 down-regulation promotes FAO low activity and, therefore, protects lipotoxic cell death. Moreover, HCC cells knocked down for CPT2 acquired resistance to lipotoxicity cell death by inhibiting FAO and Src-mediated c-jun NH-2-terminal kinase (JNK) activation [109,110,111,112]. Therefore, although FAO activity is essential to maintain the NADPH amount and ATP generation, suppling energy in several tumours [90,113,114], an excessive activity of electron transport chain may produce reactive oxygen species (ROS) and oxidative damage, leading to cancer cell death [115,116]. However, the studies regarding the role of FAO in HCC progression are extremely variable, depending on different conditions that are explained below. 

### 2.1. Oxysterols and HCC

While the implication of FA metabolism has been widely described, the direct effect of oxidised sterols in HCC development remains unknown. However, it is known that patients with HCV hepatitis and NAFLD present higher serum concentrations of oxysterols [117,118]. Accordingly, in a previous study, we reported that supplementation with cholesterol in HFD-fed mice determine the hepatic accumulation of specific oxysterols (e.g., 7β-hydroxycholesterol, 7-Ketocholesterol, and 5α-cholestane-3β,5,6β-triol), which impair mitochondrial function, acting synergistically with FAs and therefore facilitating NAFLD progression [119]. However, some typical oxysterols associated with NAFLD (e.g., 25-hydroxycholesterol) are known ligands of liver X receptor (LXR), whose low tumoral expression has been recently suggested as a negative prognostic marker in patients operated for HCC [120]. In fact, LXR enhances, at a transcriptional level, the cytostatic and pro-apoptotic effects of transforming growth factor beta (TGFβ-1) [121] and inhibits HCC cell proliferation by through activation of suppressor of cytokine signalling 3 (SOCS3) [122].

### 2.2. Lipid Metabolism in Hepatocellular Carcinoma Progression

HCC is characterised by several histological features with different underlying diseases, and, therefore, studies are not conclusive on the role of FAO. Lu et al. demonstrated that activation of FAO stimulates HCC cells to survive energy deprivation via expression of CCAAT/enhancer binding protein alpha (C/EBPα), which in turn induces autophagy [123]. Accordingly, other reports highlighted the importance of 5′ adenosine monophosphate-activated protein kinase (AMPK) in FAO enhancement in HCC when nutrients are scarce [113,124]. Moreover, ACC can modulate FAO activity in human and murine HCC as it forms a complex with carnitine palmitoyltransferase 1A (CPT1A). In a nutrient deficiency condition, AMPK phosphorylates ACC, which dissociates from CPT1A, permitting its translocation to the mitochondrial membrane to sustain FAO by FAs transport [98]. Cassim et al. proved that HCC cells show a prevalent glycolytic metabolism, but under glucose deprivation FAO is activated, supplying energy and facilitating proliferation [125]. In contrast, in the condition of hypoxia, HCC cells react differently, as FAO is repressed. When the tumoral growth rate accelerates, the blood oxygen supply could be inadequate, thereby provoking hypoxia [126]. Two recent studies propose that hypoxia inducible factor 1-α (HIF-1α) induces FAO inhibition, protecting HCC from the excessive production of ROS under hypoxic conditions [115,127]. HIF-1α reduces the expression of medium- and long-chain acyl-CoA dehydrogenases (MCAD and LCAD), two rate-limiting enzymes involved in mitochondrial FAO initiation, as demonstrated in vitro in a rodent model and human specimens [115]. Another proposed pathway of HCC adaptation during hypoxia involves the up-regulation of mitochondrial acetyl-CoAsynthetase 1 (ACSS1), which converts acetate to acetyl-CoA [91]. In this gene expression study of 361 HCC tissue, the authors structured a model to identify metabolic processes involved in tumour proliferation, predicting that in hypoxic conditions, FA synthesis is enhanced, while FAO is repressed. Furthermore, ACSS1 high expression was associated with malignancy [91]. 

Despite these reports, Iwamoto et al. described that oxygen and nutrient depletion by antiangiogenetic drugs provokes a metabolic switch to lipid-dependent metabolism by raising FA uptake and FAO [128]. Notably, in murine models of tumour implantation in a lipid-rich environment (i.e., adipose tissue and steatotic liver), the authors showed that metastatic cells can easily proliferate because of increased FA uptake and catabolism.

Another condition in which enhanced FAO permits tumoral progression is βcatenin-activated HCC. A quote of HCC develops in non-cirrhotic livers and in patients with metabolic syndrome without fibrosis [129,130,131], partially explained by an alternative mechanism for HCC development, which is called β-catenin-activated signalling. Senni et al. showed a different lipid metabolic reprogramming in βcatenin-activated HCC as the expression of PPARα and CPT2 is increased with a consequent enhancement of FAO in humans and mice [132]. PPARα is also able to up-regulate the expression of MCAD and LCAD, thereby fuelling FAO initial steps. Here, the authors corroborate data by PPARα genetic ablation and CPT1 inhibition with etomoxir, blocking development and progression in βcatenin-activated HCC in mice. In contrast, we have discussed above that in non-mutated βcatenin HCC, CPT2 is normally downregulated [91], as well as in SH-HCC and HFD-fed MUP-uPA mice [133,134]. However, studies also recently reported βcatenin activation in cases of NASH [97,135,136,137]. 

**Figure 2:**
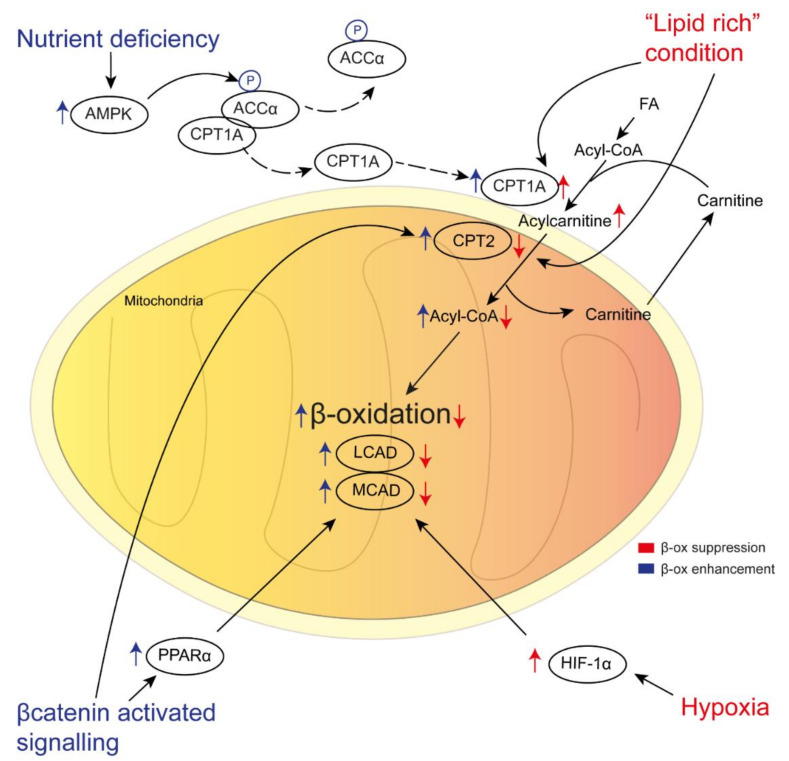
Different conditions influencing β-oxidation in HCC and proposed mechanisms. In a “lipid-rich condition” (e.g., obesity, non-alcoholic steato-hepatitis (NASH)) carnitine palmitoyltransferase 1A (CPT1A) is up-regulated, while CPT2 is down-regulated with consequent accumulation of pro-carcinogenic acylcarnitine and lower availability of acyl-CoA to sustain β-oxidation. Hypoxia has also been associated with β-oxidation suppression, as hypoxia inducible factor 1-α (HIF-1α) is induced and inhibits the expression of medium- and long-chain acyl-CoA dehydrogenases (MCAD and LCAD), two rate-limiting enzymes involved in the first β-oxidation steps. In contrast, in βcatenin-activated HCC, β-oxidation is fuelled by CPT2 activity, while peroxisome proliferator activated receptor alpha (PPARα) is up-regulated and induces the expression of LCAD and MCAD. During nutrient deficiency, 5′ adenosine monophosphate-activated protein kinase (AMPK) phosphorylates ACCα, permits CPT1A migration to the mitochondrial membrane to transport FAs and sustain β-oxidation. Fatty acid (FA); carnitine palmitoyltransferase 1A (CPT1A); carnitine palmitoyltransferase 2 (CPT2); medium-chain acyl-CoA dehydrogenases (MCAD); long-chain acyl-CoA dehydrogenases (LCAD); hypoxia inducible factor 1-α (HIF-1α); peroxisome proliferator activated receptor alpha (PPARα); 5′ adenosine monophosphate-activated protein kinase (AMPK); acetyl-CoA carboxylase alpha (ACCα).

Finally, it is intriguing to observe that, although FAO activity suppression is beneficial for HCC development, especially in obesity and NASH, increasing β-oxidation is fundamental in hypoxia, nutrient deficiency, and β-catenin-activated HCC (Figure 2). Moreover, enhancement of FAO seems to be essential in cancer stem cells (CSCs), a subpopulation of cells generally resistant to chemotherapy and able to rapidly regenerate tumours [138,139,140,141]. Although the existence of CSC in HCC is still debated, Chen et al. demonstrated that, in murine liver, CSCs, with the cell marker NANOG, modulate the expression of several mitochondrial genes (*Acadvl, Echs1*, and *Acads*), supplying energy by FAO [142].

The enzymes involved in FA de novo synthesis and mentioned in the previous paragraph can show a role in tumoral progression as well. For instance, ACC1, the first rate-limiting enzyme in de novo lipogenesis, has been associated with HCC cell survival under metabolic stress, such as glucose deprivation, accounting it as an independent predictor of poor HCC prognosis [98]. FASN is responsible for palmitate (C16:0) synthesis from malonyl-CoA and cetyl-CoA [143]. Its fundamental role in HCC has been demonstrated by genetic ablation and pharmacological inhibition, both in vitro and in vivo [92,94,144]. Moreover, in human HCC, FASN acetylation is reduced and thereby protected by proteasomal degradation [145]. Destabilization of FASN by acetylation suppresses the growth of HCC. Gene expression profile studies in patients showed that higher expression of SCD-1 and SREBP1 are associated with poorer prognosis [92,94,144], underling a role in HCC progression and resistance. Budhu et al. found that palmitoleate (C16:1), the biological product of SCD1, facilitates HCC cells migration, while the ablation of SCD1 in HCC cells diminishes migration and xenograft development [88]. Furthermore, SCD1 overexpression confers sorafenib resistance in HCC, while SCD1 knockdown makes HCC tumour initiating cells more sensitive to sorafenib via ER-stress-induced unfolded protein response [146]. Similarly, down-regulation of CPT2 has been associated not only with hepatocarcinogenesis, but also with cisplatin chemoresistance of HCC cells [147].

### 2.3. Lipidomic Contribution to HCC Progression Knowledge

As already shown, several studies tried to deepen the mechanisms by which lipid metabolism rearrangement is involved in development and progression of HCC, mostly by gene expression analysis. However, as HCC cells display a different metabolic behaviour in vitro, rather than in vivo [148], this HCC heterogeneity imposes a profound understanding of the lipidic profile of individual cells. This is possible thanks to a branch of metabolomics, the Lipidomics, which was introduced in 2003, permitting researchers to characterise the diversity of FAs and other lipids in cells, tissues, organs, and organic fluids. Some studies already showed the importance of hepatic lipid composition in NAFLD and particularly in changes of free FAs ratios [149,150]. Patterson et al. described that serum of HCC patients is enriched in glycodeoxycholate, deoxycholate 3-sulfate, bilirubin, bliverdin, and other fetal bile acids, while lignoceric acid and nervonic acid, two very long-chain fatty acids (VLCFAs), were particularly increased in plasma of HCC subjects compared to cirrhosis [151]. Accordingly, VLCFAs are involved in inflammation as lipid mediators, showing a putative role in hepatocarcinogenesis [152]. Other studies have been subsequently published some common or controversial data, which we try to elucidate in this review. Some reports agree with the observation of increased saturated and monounsaturated FAs (SFAs and MUFAs) and with the contemporary reduction of polyunsaturated FAs (PUFAs) in HCC [153,154]. This was also related to HCC severity [153] and NASH [150]. Moreover, in Pten-null mice with NASH or HCC, an increased ratio of long n6-polyunsaturated FAs to n3-polyunsaturated FAs was reported [155], while in fat-1 transgenic mouse omega-3, polyunsaturated FAs dampened inflammation and tumorigenesis [156]. Phosphatidylcholine, containing palmitoleic acid or oleic acid, was reported as elevated in HCC by Morita et al. [157], while krautbauer described a reduction of this molecule [158]. Lu et al. also found high levels of phosphatidylcholine in HCC tissue, but the aim of their research was to find different lipidomic profiles between tissue and plasma in HCC patients, proposing plasmalogens (36:4) and (40:6) as potential biomarkers of diagnosis and tumoral progression [159]. Moreover, they described higher hepatic levels of six TAGs, one sphingomyelin (SM), and one ceramide (CM). In contrast, two works reported an important reduction of CM levels in HCC tissues with a concomitant increase of SMs [153,158]. This underlines an impaired activity of sphingomyelinase with a consequent lower conversion of SM to ceramide. The alteration of sphingolipid metabolism is associated with cancer, because levels of pro-apoptotic lipid ceramide are reduced, while levels of proliferative lipids SMs are elevated [160]. Another important lipidic product is Acylcarnitine, which has been proposed as a marker of HCC diagnosis and prognosis. Particularly, two studies showed higher levels of long-chain acylcarnitines and lower levels of medium and short-chain acyl-carnitines in HCC and serum of patients [154,161]. Acylcarnitine plays a central role in cellular lipid metabolism, as it is involved in the transport of activated LCFAs into mitochondria to sustain FAO [162]. The authors explain that the high ratio long-chain acylcarnitine/short-chain acylcarnitine is likely due to the high request of β-oxidation [154]. However, we have already discussed above that the altered expression of CPT1A (up-regulated) and CPT2 (down-regulated) could be responsible of low βoxidation and accumulation of acylcarnitine. Moreover, acetylcarnitine can be converted to malonyl-CoA with consequent inhibition of CPT1 activity and reduction of βoxidation [163]. However, we could assume that lipidomics of serum/plasma is a candidate to be used for detection of biomarkers, as blood withdrawal is easy and not invasive, thus studies are arising [154,159,161,164,165,166,167]. Chen and Passos-Castilho used serum lipidomics to differentiate patients with HBV-related HCC from HBV chronic hepatitis [164,166]. Similar analysis was performed on serum with discrimination of HCV-related HCC from patients with chronic HCV [167]. Fages et al. collected blood samples from HCC patients before and after diagnosis and identified 16 metabolites, involved in lipid and aminoacids metabolism and ammonium detoxification, which can differentiate HCC patients from controls and, most notably, can predict HCC development [165]. Recently, lipid profiling in HCC cells revealed that low levels of acyl-based glycerophospholipids, an important component of the cell membrane, were associated with metastatic activity [168].

Collectively, these studies (Table 1) show that lipidomics contributes to the understanding of metabolic alteration in HCC, especially if combined with transcriptional studies, and provides potential new biomarkers of disease diagnosis and progression.

### 2.4. Lipid Zonation

In the adult liver, the hepatocyte is a highly specialized cell polarized with three different membrane domains: sinusoidal (basal), lateral (or inter-hepatocytic), and canalicular (apical). Moreover, the hepatocyte function varies widely within the lobule localization, as shown by different gene expression profile or biochemical activity [169,170,171]. Three zones in the liver sinusoid with different functions may be recognized: Zone 1 has the higher oxygen tension, as it is extended around the portal tract, receiving blood from hepatic artery and from the portal vein; zone 3 is around the portal vein, thus with a very low oxygen tension, while zone 2 is between zone 1 and zone 3. The lobular structure exposes groups of hepatocytes to different concentration of oxygen, nutrients, toxins, and intestinal-derived molecules, and, therefore, hepatocytes exhibit different metabolic functions, such as glycolysis, gluconeogenesis, and FA metabolism [172,173]. It is known that about 50% of the expression of liver genes are zonated, revealing, for example, that FAO and gluconeogenesis increase in the portal side, while lipogenesis is preponderant in the central side [170,174,175]. The zonal distribution of steatosis is not well understood yet, although it is known that steatosis is preponderant in the pericentral area and this heterogeneity is visible across the entire liver tissue [176,177]. Moreover, the severity and the localization of lipid accumulation have been correlated to NASH [178]. In this work, the authors report that, in liver biopsies from 500 patients with NAFLD, the severity of steatosis was positively associated with lobular inflammation and fibrosis in zone 3, whereas around the central veins, where oxygenation is low, a worse steatosis presented advanced fibrosis with Mallory body and ballooning. Accordingly, an association between phospholipids distribution and pro-inflammatory hepatic phenotype has been described [179]. 

Along these lines, it is plausible that distribution, storage, and metabolism of lipids change in a specific manner and in specific lobular zones, driving the progression of steatosis to NASH, cirrhosis, and cancer. The analysis of human specimens from simple steatosis revealed a zonation of expression of enzymes involved in phosphatidylcholine synthesis, confirmed by the zonation of specific phosphatidylcholines [179]. Intriguingly, in NASH biopsies, this lipid zonation is lost, identifying a potential mechanism of disease progression. Recently, Hall et al., analysing human and murine livers with advanced mass spectrometry imaging, identified several lipids with different zonations between the controls and NAFL, and notably they also observed the loss of zonation in NASH [180]. Furthermore, they asserted that the distribution of arachidonic acid-containing lipids drive inflammation in NASH pericentral hepatocytes, by releasing arachidonic acid from membranes with eicosanoids production [180]. However, HCC can occur in early stages of NAFLD without the development of cirrhosis or fibrosis, but the mechanism remains elusive [47]. One potential mechanism, described as the driver of liver tumorigenesis without fibrosis, is the activation of the β-catenin pathway [130].

Wnt/βcatenin signalling has been recently indicated as a regulator of liver function and development and is responsible for hepatic zonation [172,173]. This pathway is suppressed in the periportal area and preponderant in the pericentral area, where it modulates the expression of several genes. As already mentioned, the aberrant expression of β-catenin drives a subset of hepatic tumours [172,173], thus, 25–40% of HCC presents mutation in genes with β-catenin activation. Knockout (KO) mice and expressing transgenic (TG) mice have been used to study the implication of β-catenin signalling in lipid metabolism [181]. Here, HFD-fed TG mice showed the obesogenic phenotype with predominant pericentral steatosis. In addition, expression of glycolytic and lipogenic genes was higher in HFD-fed TG mice than in KO mice [181]. In another study, mice with hepatocyte overexpression of HCV proteins (FL-N/35 model) presented a zonated pattern of lipids, as the lipid accumulation occurred in a couple of hepatocyte rows in the middle zone of the hepatic lobule [182]. In addition, the expression of FASN and other enzymes, such as SCD-1 and acyl-CoA synthase long-chain family member 3 (ACSL3), were increased. This particular zonation profile was validated in 50 human biopsies of HCV-infected patients. Notably, the authors found the up-regulation of genes involved in the wnt/β-catenin pathway in mice and in higher levels of nuclear β-catenin in human livers. Accordingly, Edamoto et al. documented wnt/β-catenin signalling involvement in HCV-related HCC [183]. Overall, wnt/β-catenin plays a key-role in lipid metabolism and zonation; however, the mechanism by which this is linked to tumorigenesis in the liver is not clear and further studies are eagerly needed.

## 3. Conclusions

HCC is a very heterogenous tumour with many factors involved in pathogenesis and different local environmental conditions; therefore, finding new and safe molecular therapy is complicated. Although new antiangiogenetic drugs and checkpoint inhibitors have been approved as molecular treatments in HCC [184], the benefits in survival rate are limited. In the present review, we showed the centrality of lipid metabolic reprogramming in HCC, indicating potential therapeutic targets. Most of the described pathways are universally altered in HCC, while other genes might be targeted in specific environmental conditions, in a personalised manner. For instance, we have shown how FA synthesis is typically enhanced, while β-oxidation is heterogeneously modulated. Moreover, the lipidomics approach can integrate gene expression analysis, providing better knowledge of tumoral metabolic alterations with identification of potential biomarkers for diagnosis and prognosis. Lipidomics and gene expression data can be further combined with new technology imaging in order to define the zonation of lipid metabolic alterations. We believe that this multiple approach is essential for developing new therapies and efficient strategies in prevention and early detection of HCC.

## Figures and Tables

**Table 1:** Main lipid composition alterations in HCC revealed by Lipidomics studies. Saturated fatty acid (SFA); monounsaturated fatty acid (MUFA); polyunsaturated fatty acid (PUFA).

Lipids	Alteration	Study Reference
SFA and MUFA	↑	[149,150]
PUFA	↓	
long n6-polyunsaturated FAs/n3-polyunsaturated FAs ratio	↑	[151]
omega-3 polyunsaturated FAs	↑	[152]
Phosphatidylcholine	↑	[153,155]
	↓	[154]
Ceramides	↓	[149,154]
	↑ (one)	[155]
Sphingomyelins	↑	[149,154]
	↑ (one)	[155]
Long-chain acylcarnitines	↑	[150,157]
Medium- and short-chain acylcarnitines	↓	

## Abbreviations

Hepatocellular carcinoma (HCC); hepatitis b virus (HBV); hepatitis c virus (HCV); non-alcoholic fatty liver disease (NAFLD); non-alcoholic steato-hepatitis (NASH); phosphate cytidylyltransferase 1 choline alpha (PCYT1A); choline kinase alpha (CHKA); vascular endothelial growth factor (VEGF); very low density lipoprotein (VLDL); triglycerides (TAG); sterol regulatory element-binding protein1 (SREBP1c); acetyl-CoA carboxylase (ACC); stearoyl-CoA desaturase (SCD); fatty acid synthase (FASN); direct-acting antivirals (DAAs); fatty acid (FA); alcohol related liver disease (ALD); alcoholic hepatitis (AH); phosphatidic acid phosphohydrolase (PAP); peroxisome proliferator activated receptor alpha (PPARα); fatty acid β-oxidation (FAO); tricarboxylic acid (TCA); ATP citrate lyase (ACLY); monounsaturated fatty acid (MUFA); peroxisome proliferator-activated receptor-γ coactivator beta (PGC-1β); steatohepatitic HCC (SH-HCC); diethylnitrosamine (DEN); high fat diet (HFD); carnitine palmitoyltransferase 1A (CPT1A); carnitine palmitoyltransferase 2 (CPT2); phosphatidylinositol-4,5-bisphosphate 3-kinase catalytic subunit alpha (PIK3CA); Src-mediated c-jun NH-2-terminal kinase (JNK); reactive oxygen species (ROS); liver X receptor (LXR); transforming growth factor beta (TGFβ); suppressor of cytokine signalling 3 (SOCS3); CCAAT/enhancer binding protein alpha (C/EBPα); 5′ adenosine monophosphate-activated protein kinase (AMPK); hypoxia inducible factor 1 subunit alpha (HIF-1α); medium-chain acyl-CoA dehydrogenases (MCAD); long-chain acyl-CoA dehydrogenases (LCAD); mitochondrial acetyl-CoAsynthetase 1 (ACSS1); cancer stem cell (CSC); very long-chain acyl-CoA dehydrogenase (Acadvl); enoyl-CoA hydratase short chain 1 (Echs1); short-chain acyl-CoA dehydrogenase (Acads); saturated fatty acid (SFA); polyunsaturated fatty acid (PUFA); ceramide (CM); sphingomyeline (SM); very long chain fatty acid (VLCFA); long chain fatty acid (LCFA).
